# Toward Characterising the Cellular 3D-Proteome

**DOI:** 10.3389/fbinf.2021.598878

**Published:** 2021-03-29

**Authors:** Arne Elofsson

**Affiliations:** Department of Biochemistry and Biophysics and Science for Life Laboratory, Stockholm University, Stockholm, Sweden

**Keywords:** protein bioinformatics, protein-protein docking, deep learning, proteomics, direct couplin analysis, coevolution

## Introduction

Proteins are the central machines of cells, and they perform their actions by interacting with each other as well as with other molecules. Today, large-scale efforts in genomics, proteomics, lipidomics and metabolomics are producing complete lists of the molecules in a cell as well as in different subcellular compartments, including the membrane. Further, detailed knowledge of composition (splice forms, PTMs) and expression levels in different cells are becoming available.

However, proteins do not act on their own but by interacting with other proteins. The interactions are of many different types, from very stable interactions in large protein complexes to transient interactions by disordered regions containing linear motifs (Palopoli et al., 2020). These protein-protein interactions can be studied at different levels of detail. Only for a small number of the large complexes atomistic the structure is known and in particular molecular complexes embedded in the membrane are challenging to study experimentally. Today Cryo-EM provides high-resolution structural information for many large biological complexes.

However, experimental techniques are not applicable for all type of protein interactions, as many biological interactions are transient and contain weakly interacting proteins. These complexes are virtually impossible to purify or crystallize and therefore for many biological complexes no or only low-resolution structural information is available. Here, only computational methods will be able to provide detailed structural information, but also for the stable complexes computational methods will be of great importance.

Computational methods to predict the structure of individual proteins or protein complexes have, until recently, almost exclusively been based on homology transfer. Here, structural information is transferred from one protein to another, assuming that the structure if homologous proteins (or complexes) is conserved. However, today by using co-evolution and advances in deep-learning, it is now possible to predict the structure of many individual proteins and complexes directly using no other information than the sequences and their evolutionary history. Below we will briefly describe, the past, present and future of these type of methods.

## Structure Prediction by Co-Evolution and Deep Learning

The basis for the progress in protein structure prediction is the development of contact predictions methods using direct coupling information. The predicted contacts can then be used to predict the structure of individual protein (Marks et al., 2011) as well as of protein-protein interactions (Weigt et al., 2009). One significant limitation of these methods is that they can only be applied on very large protein families. Deep learning methods have been developed to overcome this problem (Skwark et al., 2014; Wang et al., 2017). Lately, by changing the problem from predicting contacts to predicting distances, another leap in performance has been obtained (Xu, 2019; Senior et al., 2020).

### The Challenges

In short, a complete 3D-proteome would require that the following subproblems are solved.

1. Structure prediction of individual proteins.
2. Identification of what molecular components interact.
3. Predicting the structure of interacting molecules.

#### Structure Prediction of Individual Proteins

The progress of the prediction of individual proteins has been tremendous in the last decade. The idea to use co-evolution to predict contacts has been around since the mid-1990s, but until ten years ago, the success was minimal. Then the direct coupling analysis (DCA) methods were introduced, and everything changed. In the first DCA studies, only protein families with more than a thousand sequences could be predicted accurately. Now often accurate predictions can be obtained for much smaller families, and besides, the families have grown.

The last CASP meetings have reported a leap in performance for difficult protein structure prediction targets showing that ab-initio structure prediction is accurate for most proteins (Kryshtafovych et al., 2019). Initial attempts to predict the structure of all protein families, such as PconsFam (Lamb et al., 2019) predicted the structure of a few hundred Pfam families. However, the recent progress should multiply this number, and today it is possible with high confidence to predict the structure of all but ten proteins in a minimal genome (Greener et al., 2020), indicating that the structure of most protein domain families can be predicted already today.

However, large parts of the human proteome lack domains this sometimes referred to as the dark proteome (Perdigao et al., 2015). We have shown that these regions are both longer and more disordered in eukaryotes than in prokaryotes (Basile et al., 2019). This indicates that it is not only a lack of data that separates these regions from the ones assigned to domains. Therefore, it remains to be studied in detail how the (potential) structure of these regions can be predicted although some attempts have been made (Toth-Petroczy et al., 2016).

#### Identification of Molecular Interactions

Interaction between proteins can be of many different types. Some proteins are tightly bound together in a molecular machinery, while others interact only transiently. By a combination of large-scale studies using methods, the general properties of interaction networks of proteins are rather well understood. For instance, the analysis of these networks has shown that proteins with many interactions often contain long disordered regions or domain repeats (Ekman et al., 2006). However, the exact knowledge of most protein interactions is missing.

Large scale information on how proteins interact is obtained from various experimental methods including, yeast two-hybrid, tap-tag, and co-expression. However, other information such as gene-fusion, and genome localization, can also contribute to the identification of interacting protein pairs. Therefore, by combining experimental and, bioinformatical data insights can be gained (von Mering et al., 2005). Unfortunately, most of the large scale methods are quite noisy and prone to both false positives and negatives. However, it is possible that structural modeling can help to remove some of the noise (Cong et al., 2019).

For proteins to interact, they do need to exist in the vicinity of each other, i.e., be located in the same subcellular compartment. Although subcellular localization of proteins has been studied for decades, it is still not at all clear in what compartment many proteins exist. For instance, recent studies indicate that many proteins are found in the nucleus than previously observed (Stadler et al., 2013). Therefore, improved prediction of subcellular localization would help to identify interacting proteins (Almagro Armenteros et al., 2017).

Currently, the only docking method that has been shown to provide any useful information if two proteins interact or not is template-based docking. In 2012 Honig and co-workers used PrePPI to estimate if protein pairs interacted or not showing that a purely computational method could be as efficient as the experimental methods (Zhang et al., 2012). This method has later been improved (Mirabello and Wallner, 2017).

Many proteins, in particular in eukaryotes, have closely related paralogs. These often do not have the same interaction partners. To distinguish which paralog interacts with another protein is often not possible using template-based docking, as the paralogs all are quite similar. Here, co-evolution can aid. Bitbol showed that it is possible to identify the correct interacting pairs within the bacterial two-component system using co-evolution and a maximum entropy approach (Bitbol et al., 2016).

#### Modeling the Structure of Interacting Proteins

In addition to detecting if two proteins interact it is important to model how they interact. Protein-protein docking has, for a long time, been a challenge for computational biology (Porter et al., 2019). Although some progress has occurred in this field the CAPRI evaluations has not seen the same progress as in CASP (Wodak et al., 2020). Possibly this is due to the lack of participants using co-evolutionary methods.

If two proteins have perfectly complementary surfaces, protein-protein docking is trivial. However, this is rarely the case as structural plasticity of the involved proteins that change the interaction surfaces. Further, the use of modeled structures change the interaction surfaces even more, making the practical use for protein-protein docking methodologies to be of limited practical use. Therefore, often it has been necessary to use low-resolution experimental information to obtain reliable results as we did when predicting the structure of the Tom-complex in mitochondria (Imai et al., 2013).

Recently an alternative method, template-based docking, has gained popularity (Zhang et al., 2012). Here, two proteins are not docked given all degrees of freedom, instead, the database of known protein complexes are used as templates to guide the docking, in a similar fashion as templates is used in homology modeling. Template-based docking is useful both for the identification of protein interactions and for determining the structure of the complex (Kundrotas and Vakser, 2013).

The use of the same direct-information contact prediction methods as described above can be applied for protein-protein docking (Schug et al., 2009; Hopf et al., 2014; Ovchinnikov et al., 2014). In theory, these methods can be used without modification to obtain contact information between proteins pairs. However, to predict inter protein contacts, it is necessary to identify the exact pairs of proteins that interact. The problem is that even if it is known that protein *A* and *B* interact in one organism and that both proteins have homologs $A^{\text{*}}$ and $B^{\text{*}}$ in another organism it is not certain that $A^{\text{*}}$ and $B^{\text{*}}$ interact. For instance, $A^{\text{*}}$ might interact with a paralog $B^{\text{**}}$ or the interaction might just not be conserved between organisms. Further, homo-multimers also cause problems for the current set of methodologies. Therefore, improved methods for generating correct multiple sequence alignments as well as improved contact predictions methods are probably necessary to develop.

### Status of Docking Methodologies

**FIGURE 1:**
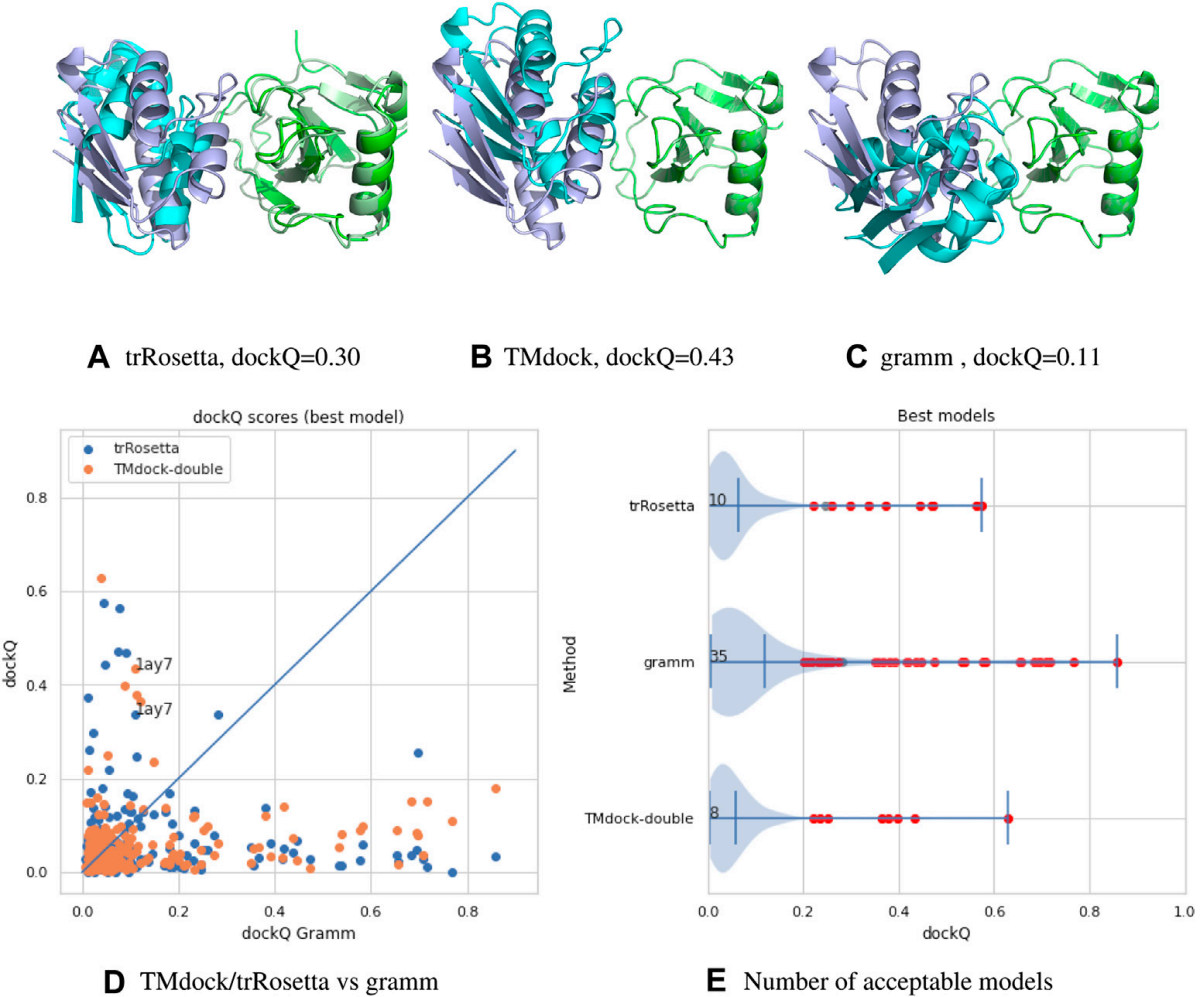
**(A–C)** comparison of models for target 1ay7 (the model with the best median score) generated by three different docking protocols, trRosetta (contact-based docking), TMdock (template-based docking) and gramm (free docking). All superpositions is done on the first protein chain (in green). The native pdb structure is shown in lighter colors. For trRosetta both chains are shown as they are both folded, while for the other methods the first chain is identical to the native structure and therefore not seen. **(D,A)** A comparison of docking qualities for 218 heterodimeric proteins pairs from the dockground 4.3 dataset using dockQ (Basu and Wallner, 2016) for the three methods. A dockQ score of 0.2 or higher is often used as an indication for an acceptable docking model. The number represent the number of models a certain method is better than the other two and that the quality is higher than this cutoff. For each target the score for the best of the top-10 ranked modes is shown.

A comparison between the three methods can be found in Figure 1. Here we compare three different docking strategies on a common dataset of unbound structures (Kundrotas et al., 2018). Gramm (Vakser, 1995) is a state-of-the-art free docking tool. Models are generated by shape complementary and ranked according to a potential function. TMdock (Kundrotas amd Vakser, 2013) is template-based docking program. Here we excluded all hits where both proteins have highly homologous proteins in PDB(Westbrook et al., 2002) (E-value < 1. e-5). Finally, we have used trRosetta (Yang et al., 2020), a contact-based structure prediction method, to dock two proteins. Here we modified the protocol so that both proteins are folded and docked simultaneously. For each of the two proteins, multiple sequence alignments were generated using jackhmmer (Finn et al., 2011) over representative proteomes from UniProt (The UniProt Consortium, 2017). The two MSAs were then merged by hits found in the same proteome for both sequences. We used several different alignment strategies, including different E-value cutoffs and attempts to identify orthologs using reciprocal best hits. For all three methods, ten models were generated, and the best, according to dockQ, was used. No significant difference is seen if only the top-ranked model is used.

It is clear from this brief analysis that 1) for the majority (165/218) of protein no method provides an accurate model 2) still traditional docking methods provide the highest number of acceptable models (Xu, 2019) 3) the methods are complementary to each other (only for four targets two methods provide acceptable models).

## Deciphering Cellular Networks

Cells consists of complex networks of interacting biomolecules. In addition to the physical-stable interactions between proteins that I have discussed above other regulatory interactions are important. These includes miRNA bases regulations, proteins binding to promoter regions, and many other type of interactions. Many if these are studied and predicted in databases such as string (von Mering et al., 2007).

Although all interactions are of great interest, it is unlikely that the revolution seen by deep learning and DCA will equally affect our ability to predict all types of interactions. The first requirement is that we have sequence data for both molecules interacting and that there is an evolutionary pressure for these to co-evolve. This excludes all interactions including anything but RNA, DNA and proteins. However, it has been shown that co-evolutionary signals also can be used to detect epistatic interactions in bacteria (Skwark et al., 2017) and viruses (Zeng et al., 2020). However, one should not forget that at the end there is always a physical interaction underlying all types of interactions.

## Future Outlook

Complete structural knowledge of all proteins and their interactions in a cell will change our understanding of cell biology in the same way as the human genome project changed our understanding of genetics. It is not only a question about knowing what is there but also what is missing, i.e. what interactions do not exist. Given the rapid progress in both experimental and computational methods, this is no any longer an unrealistic scenario. During the revision of this paper Deepmind presented their impressive results at the CASP14 conference, strengthening the assumption that the prediction of the structure of stable protein domains is basically a solved problem and that one of the focus should be on how proteins interact in the cell.

Clearly the interaction data provided from computational methods needs to be complemented with experimental data. Here, one has to distinguish between transient and permanent interactions. Cryo-EM tomography will provide very valuable low-resolution data for permanent interactions, but not for transient interactions.

Transient interactions are fundamental for regulation in a cell. These interactions can be of different natures, from the folding-upon-binding type of disordered regions to rigid body interactions to phase transitions in cellular bodies. The evolutionary pressure on these interactions is also different making it difficult to use co-evolutionary methods for some type of interactions. For instance, the binding of disordered motifs often shows no co-evolutionary signal, as the disordered motif is extremely variable, while the interaction surface is very conserved.

Anyhow, we believe that results from the rapid advancement in structure prediction show the power of machine learning methods. Therefore, this author is convinced that it is just a question of time until these methods (based on co-evolution) will be applied to predict interactions on a large scale. Further, I believe that the main problem in the short time will be to construct the optimal multiple sequence alignments for detecting the interactions.
